# Analysis of influencing factors of subjective career unsuccessfulness of vocational college graduates from the Department of Navigation in China

**DOI:** 10.3389/fpsyg.2022.1015190

**Published:** 2022-11-22

**Authors:** Li Wang, Jian-Hong Ye, Yi-Sang Lee, Cong-Jin Miao

**Affiliations:** ^1^Dhurakij Pundit University, Bangkok, Thailand; ^2^Dean's Office, Hainan Vocational University of Science and Technology, Hainan, China; ^3^Faculty of Education, Beijing Normal University, Beijing, China; ^4^Department of Industrial Education, National Taiwan Normal University, Taipei City, Taiwan; ^5^Faculty of Maritime, Hainan Vocational University of Science and Technology, Hainan, China

**Keywords:** adaptive action, adaptive preparation, career adaptability, career construction, situation, Situational expectation value theory, subjective career unsuccessfulness, vocational education

## Abstract

The marine talent cultivation and output in higher vocational colleges is an important support to build a strong maritime country and ensure the steady development of the shipping business industry. Vocational colleges should ensure effective career preparation and career guidance education for their students, and train them to acquire the professional abilities to work and adapt effectively and quickly in the future. Some studies show that many crew members experience a decline in job satisfaction, a low sense of achievement, and a series of negative subjective career feelings. Even more, some crew members have poor work performance or unsuccessful career development behaviors such as career change or resignation. This study examined the causes and influencing factors of these circumstances and might be a reference for schools to strengthen the quality of their training programs in the future. In-depth interviews with 12 vocational marine navigation college graduates were conducted, and grounded theory was used to code and analyze the collected interview information. Four types of influencing factors were identified: adaptation preparation, career adaptability, adaptation action, and the situational factor. The adaptation preparation factor could be categorized into psychology, determination, lack of certificates, and simple work; the career adaptability factor could be categorized into boring work, busyness, a big gap between expectations and reality, danger, promotion difficulty, poor interpersonal relationships, high pressure, and maladaptation to the surroundings; the adaptation action factor could be categorized into passive fatigue, lack of training, competition, confidence, and planning; while the situational factor could be categorized into the impact of the epidemic, low social status, lack of support from family, and advantages of living on land. To help the marine navigation students better adapt to their jobs and avoid subjective career unsuccessfulness, higher vocational colleges need to strengthen students’ mental health education, consolidate knowledge and skills training, encourage students to obtain more vocational competency certificates, enhance their interpersonal communication skills, do effective career planning, pay attention to physical exercise and safety awareness training, and adopt other career management and counseling suggestions.

## Introduction

The cultivation and exportation of marine talent by higher vocational colleges is considered as a significant support to build an ocean power country, ensure the stability of the shipping business industry (Bao et al., 2021), and maintain the smooth development of global trade (Beukelaer, 2021). Vocational colleges should ensure effective career preparation and career guidance education for marine navigation students so that these students will have professional skills and be able to quickly to adapt to jobs in the future. The population of Chinese crew members ranks second in the world, accounting for 11% of the total toilers of the global sea (Drewry Shipping Consultants (Drewry), 2018). Knowing their feelings about their careers and their experiences could help to understand the relative and effective operation of the shipping industry, and also provide a strong reference for the career management and counseling education of marine navigation students. An et al. (2020) conducted a survey of 337 seafarers in China and found that they often showed negative attitudes towards their work. Seafarers work long hours, with little free time, on moving, closed, and noisy ships that cause physical and mental exhaustion (Shan and Neis, 2020). With high levels of stress, crew job satisfaction drops sharply (Carlson et al., 2019). Besides, crew members are unable to obtain a balance between career and family, resulting in a decline in work motivation and even a strong inclination to quit (Bao et al., 2021). In the past 2 years, many countries have closed their borders to prevent the spread of COVID-19, creating a crew change crisis, forcing seafarers to continue working, increasing their mental stress, and resulting in worse well-being and mental health (Beukelaer, 2021). Thus, it is particularly urgent to discuss the causes of seafarers’ poor career adaptation and negative career experience.

Subjective career success refers to the positive psychological outcome of an individual’s subjective evaluation of self-work experience or career (Arthur et al., 2005). Numerous studies have discussed subjective career success (Blokker et al., 2019). For example, if individuals are satisfied with their current work status, salary, job promotion, and development according to their expectations, they will obtain a higher sense of achievement from their work (Seibert et al., 1999; Arthur et al., 2005). In addition, Zhou and Sun (2010) pointed out that satisfaction with external material rewards, position of power, exertion of talents, recognition by the organization, maintenance of physical and mental health, and balance between work and family can also be recognized as criteria for subjective career success. However, some employees are often dissatisfied with their jobs and remuneration packages, especially when the work is overloaded and there is high work pressure, which can eventually lead to turnover intentions (Yong, 2018; Pandey et al., 2021).

The data released by the Chinese Ministry of Transport (CMOT) show that the average available Chinese Seafarer labor dropped about 6% from 2016 to 2020, and less than 30% of graduates from 2017 to 2019 were engaged in seafaring work (Chinese Ministry of Transport (CMOT), 2016; CMOT, 2017, 2018, 2019, 2020). According to the 2021 annual report of “Chinese Seafarers Supply and Demand Index, CSSD,” due to the influence of Covid-19, the supply and demand relationship of seafarers in the international sailing area in 2020 dropped significantly compared with the previous year; the proportion of active seafarers with annual service qualifications in the international sailing area dropped from about 50 to 30%, showing a general shortage (China International Maritime Research Center, 2021). The attractiveness of seafaring work continues to weaken, and crew members have felt negative about their work in recent years. Therefore, sorting out the causes of seafarers’ subjective career failure is crucial to solve the problems of career loss, to stabilize industry development, and to promote career management and counseling education for seafaring students.

Byrnes et al. (2020) adopted quantitative research methods to explain the effect of the COVID-19 pandemic on medical student career perceptions. Guan et al. (2017) used experimental research methods to represent the relationship between futurework self, occupational adaptability, and occupational exploration. Zeng et al. (2022) surveyed 636 Chinese vocational high school students to quantify the relationship between career adaptability and academic self-efficacy as hope, futurework self, and life satisfaction. Şahin and Gülşen (2022) conducted a quantitative study of undergraduate students and showed that self-leadership played a partial mediating role between satisfaction with basic psychological needs, academic self-efficacy, and career adaptability. Previous studies on the career development of students mainly adopted quantitative rather than qualitative methods.

Qualitative research emphasizes that researchers go deeply into social phenomena, understand the thoughts of the observed objects through researchers’ personal experience, and establish a “contextualized” and “intersubjective” meaningful interpretation on the basis of collecting original data. Quantitative research is a research method that measures and analyzes the quantifiable part of a thing to test some theoretical assumptions of the researcher (Chen, 2000). On the contrary, qualitative research focuses more on microscopic or special phenomena of the interactive experience between the researcher and the observed objects. From the perspective of the observed objects, based on the original phenomenon itself, through continuous comparison, reflection and induction, a more overall comprehensive understanding and explanation is acquired. The use of qualitative research is based on a certain situation when there is no predetermined hypothesis on the understanding of the observed objects. Therefore, it is very important to obtain more comprehensive and newer research results.

Unlike those studies, this study adopted a qualitative method, in-depth interviews with marine navigation graduates, to understand the graduates’ subjective career feelings about their adaptation and integration with people and their surroundings, and the process of career construction. Career construction is very important to relative occupation studies (Savickas, 2002, 2005). Meanwhile, with the assimilation in the current situation, the situational expectation value theory emphasizes the situation, cultural background, expectations, personal choices, behaviors, and performance, to explain the causes of graduates’ subjective feelings (Eccles and Wigfield, 2020). This study aimed to provide suggestions to marine navigation students in higher vocational colleges for their career management and education in the future. As a result, this study focused on what influenced the connotation of the subjective career unsuccessfulness of higher vocational marine navigation graduates.

## Theoretical background

### Career construction theory

Career Construction Theory (CCT; Savickas, 2002, 2005) is one of the most important occupation research theories (Akkermans et al., 2020). The core concept is to adapt to the social environment and achieve the goal of interaction and integration between people and the environment. This theory focuses on the process of career construction emphasizing the adaptation preparation which affects career adaptability, adaptation actions, and lastly adaptation results. Situational factors moderate and affect all links in the construction process (Savickas and Porfeli, 2012). Many scholars study career development, job satisfaction, career success, and subjective career success as positive adaptive outcomes based on the career construction theory (Rudolph et al., 2017; Haibo et al., 2018; Kim and Shin, 2020).

However, subjective career unsuccessfulness can be interpreted as a negative adaptive result. Based on the perspective of career construction theory, there are many negative factors in the construction process which make career construction difficult. This challenging process causes poor career adaptation, negative career experience, and subjective career unsuccessfulness. Career construction theory focuses on the whole process of career adaptation and takes into account the influence of situational conditions and psychological changes. The qualitative research on the subjective career unsuccessfulness of nautical graduates could explain the phenomenon more comprehensively and theoretically. It would be helpful for researchers to categorize the numerous influencing factors into adaptation preparation, career adaptability, adaptive action, and situational factors. Furthermore, researchers could redefine subjective career unsuccessfulness for the nautical graduates in this study.

### Situational expectation value theory

Situated Expectancy Value Theory (SEVT) is derived from the extension of Expectancy Value Theory (EVT) proposed by Eccles and Wigfield (2020). EVT indicates that the individual’s expectations and value judgments on work tasks determine their choice, persistence, and performance of achievement behaviors. SEVT emphasizes the influence of situational and cultural context on personal development expectations and values. Kirk-Johnson et al. (2019) adopted EVT to explain that people become involved in certain activities based on their personal value consideration. Sun et al. (in press) used SEVT theory to explain that college students’ participation in online learning and their degree of autonomous learning are determined by their perceptions of the value of the learning goals. Kuhn et al. (2022) used EVT to illustrate that the combined effects of expectations, value beliefs, and socialization determine whether teachers choose to become mentors. Wu et al. (2020) emphasized that the level of engineering self-efficacy and subjective task value positively affects the strength of achievement behavior. Therefore, this study examined how the observed subjects made certain choices, persisted, and performed in the process of career construction through the consideration of expectations and values in different situations according to the Situational Expectation Value Theory. These choices, persistence, and performance might be adaptation preparation, career adaptability, adaptation actions, or even adaptation results in career construction. Similarly, these choices, persistence, and performance might also be the psychological tendency, psychological construction, and psychological context of nautical graduates. Through this theory, the relationship between situational factors and a link with career construction was established. The situational factors were effectively linked to adjust and affect the career construction process to explain more about that process for nautical graduates.

### Definition of nouns

#### Adaptation preparation

Adaptive preparation refers to the subjective willingness or readiness to adapt (Savickas and Porfeli, 2012). Insufficient adaptation preparation will lead to poor career adaptation. Research shows that when teachers change their workplaces, if they lack experience and psychological preparation, they may eventually choose to leave due to poor adaptation and inability to cope with environmental changes (Johnson and Birkeland, 2003). DeAngelis et al. (2013) also mentioned that teachers with insufficient preparation are more likely to quit school. Moreover, some studies have also mentioned that distrust in oneself, lack of determination and courage, and negative personality activity characteristics together with insufficient willingness to adapt can affect the successful development of one’s career (Lamanauskas and Augienė, 2014). Adaptive preparation in this study refers to a negative state of weak willingness to adapt to seafaring work or poor readiness, manifested as insufficient psychological preparation, determination, certificates, and cognitive practice work.

#### Career adaptability

Career adaptability is a mental ability to adapt to career changes and to interact with the environment cognitively, behaviorally, and emotionally. Individuals pay attention to the future of their careers, take control of their career development, develop various skills, look forward to future performance, and increase their confidence in their ability to realize their aspirations. All of the above indicate that individuals with strong career adaptability are able to enhance their awareness of the future to meet higher expectations (Savickas and Porfeli, 2012; Nikander et al., 2022). On the contrary, individuals who show weaker career adaptability have stronger negative feelings of exhaustion and disengagement from work if their future career development is hindered, if difficulties arise, if a gap exists between the actual work situation and their personal expectations, if the intensity, time, and danger of the work cannot be controlled by the individual, and if the perceived self-support of the work is low (Collie et al., 2018). The lower the career adaptability, the less able the individual is to cope with changes in their career and the less happy they are (Lee et al., 2021; Waldeck et al., 2021). Career adaptability in this study refers to the psychological ability of graduates to adapt to the marine environment, including recognition of seafarer work, marine behavior, and emotional adjustment.

#### Adaptation actions

Adaptive actions refer to specific stress responses, decisions, or behaviors that are driven by career self-adjustment abilities (Savickas and Porfeli, 2012). Colarelli and Bishop (1990) pointed out that some people are willing to put in more effort and perseverance to overcome difficulties to adapt to work goals by finding professional vitality, persevering in their work, and making long-term plans (Nikander et al., 2022). Lack of planning, negative burnout, and lack of confidence are not conducive to the achievement of work goals and lead to weaker adaptive actions. The adaptive actions of this study refer to the negative reactions, decisions, and behaviors of marine navigation graduates after completing self-adjustment in adapting to marine work.

#### Situational factors

Situation refers to the relative or various combined situations within a certain period of time. Each link of the career construction process is mediated by situational factors (Savickas and Porfeli, 2012). The situational factors of this study refer to four aspects: adverse effects of the epidemic, low social status, lack of support from family members, and the advantages of land work. Due to the impact of the COVID-19 epidemic, many countries closed their borders, resulting in a “crew change crisis,” which forced seafarers on board to continue working, resulting in great mental stress and reduced happiness (Beukelaer, 2021). Emotional attention from the public to a specific occupation is a kind of social support (Carlson and Perrewé, 1999). The lower the social status, the less emotional attention and the lower social support from the public. This reflects negatively on the influence of occupational adaptability or turnover intention (Lee et al., 2021). Ryan and Deci (2000) mentioned that when an individual believes that their career development is controlled by the outside world, the external motivation will weaken their internal motivation. In other words, the more obvious the advantages of land work, the stronger the external motivation of marine graduates to give up their marine careers.

#### Adaptation results

The adaptation result is the result of the interaction and integration between the individual and the environment. The adaptive result of this study is that the graduates are not well integrated with the maritime work environment, resulting in negative personal career feelings, manifesting as subjective career unsuccessfulness. Research shows that work is fulfilling when salary, job promotion and development, and work-family balance meet one’s expectations for evaluating subjective career success (Seibert et al., 1999; Arthur et al., 2005; Zhou et al., 2013). Likewise, the inverse of the above conditions can be used to evaluate subjective career unsuccessfulness.

## Research methods

### Research method and framework

**Figure 1:**
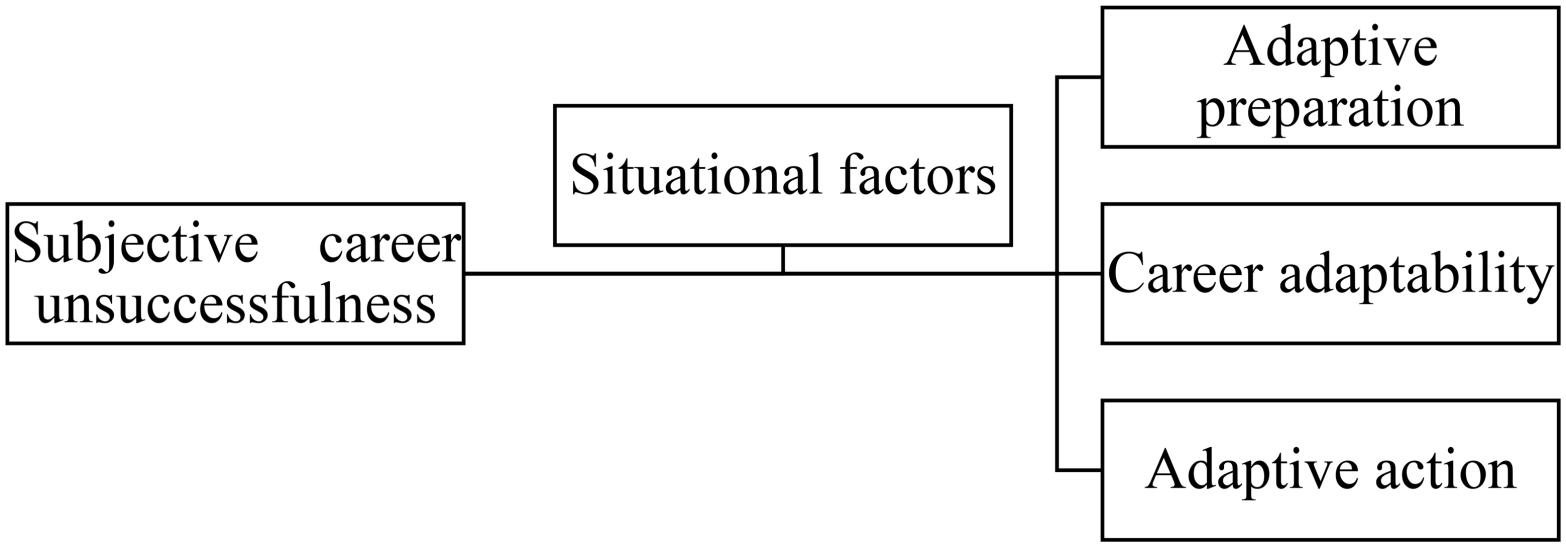
Research framework of influencing factors of subjective career unsuccessfulness.

This study examined the connotations and influencing factors of subjective career unsuccessfulness of marine navigation graduates based on the career construction theory, and conducted in-depth interviews based on grounded theoretical analysis. This study also defined the adaptive outcome of career construction as the subjective career unsuccessfulness of marine graduates, which corresponds to the main category of grounded theoretical analysis. The adaptive preparation, career adaptability, adaptive action, and situational factors of the career construction process correspond to the subcategories of grounded theory. The research framework is shown in Figure 1.

### Study conduct and participants

This study used intentional sampling to determine the heterogeneous interview samples on the basis of comprehensive consideration of different factors such as major, graduation time, and accumulated time as a seafarer in the selected vocational undergraduate college in China. Technical and Marine Engineering Technology graduates were interviewed. The basic information of the participants is shown in Table 1.

**Table 1:** Basic information of participants.

Participants	Gender	Profession	Time on board	Participants	Gender	Profession	Time on board
01	male	Navigation technology	11 M	07	male	Navigation technology	4 M
02	male	Marine engineering technology	3 M	08	male	Navigation technology	30 M
03	male	Navigation technology	6 M	09	male	Marine engineering technology	48 M
04	male	Marine engineering technology	16 M	10	male	Marine engineering technology	20 M
05	male	Marine engineering technology	30 M	11	male	Navigation technology	6 M
06	male	Marine engineering technology	32 M	12	male	Navigation technology	12 M

M = month.

In-depth interviews were conducted with 12 respondents by telephone or WeChat Voice. Before starting each interview, the interviewers briefly introduced the research and issued an informed consent form to the participants. The informed consent form stated the research process, the privacy rights of the participants, and the possible use of the subsequent research. It also reminded participants that the interview process and other matters would be recorded. The interviews were formally conducted after the participants gave their consent to participate by signing the informed consent form. During the interviews, sufficient open response time was given for the participants to stimulate discussion, which contributed to the authenticity of the information input during the 12 interviews that were all recorded.

### Research tools

Data were collected through in-depth interviews. Based on the literature review, the interview question outline shown in Table 2 was designed according to the career construction process. Questions 1–2 corresponded to adaptation preparation; questions 3–5 corresponded to career adaptability; questions 6–7 corresponded to adaptive actions; questions 8–10 corresponded to adaptation results; question 11 corresponded to situational factors; and question 12 was an open-ended question formulated specifically by the author to determine whether the participant’s career was successful or not and to determine their personal subjective feelings. Questions 1, 2, 6, 7, and 11 referred to the descriptions of adaptation preparation, adaptive action, and situational factors by Lin et al. (2007). Questions 3, 4, and 5 referred to the descriptions of career adaptability by Zhao and Guo (2010). Questions 8, 9, and 10 referred to the descriptions of adaptation outcome (career success) by Jiang and Zhou (2021).

**Table 2:** Outline of the interview on influencing factors of subjective career failure.

No.	Questions
1	What is your current job description? What personal characteristics and abilities do you think you need to acquire? How did you prepare for the job?
2	What has motivated you to work hard in these 3 years?
3	What expectations do you have for your career? What choices have you made?
4	What influencing factors do you believe will affect your future career development?
5	Do you have enough confidence in your career development?
6	Have you seen anything done by your supervisors/coworkers that is worth imitating? How can you improve your competitiveness?
7	What are your work plans for the next 3 years? What are you going to do?
8	Do you like your current work environment? Are you satisfied with your current salary?
9	Are you interested in your current job? What is the happiest thing for you in your work? What is your job achievement?
10	Can you handle job and family at the same time?Can you still enjoy life besides work? Can your job, family, and life reach a state of balance?
11	Are you facing any struggles or difficulties in your job? Are there any impressive incidents? How are you handling these situations?
12	What kinds of changes have happened in your life?Do you think of your career as successful? What are your criteria to be successful? Do you consider yourself to be unsuccessful?

### Encoding method

Grounded Theory proposed by Glaser and Strauss (1967) is a set of methods for bottom-up induction and theory construction from original data. The grounded theory research method used in this study was mainly chosen for the following reasons: 1: there were few studies on subjective career unsuccessfulness, so only in-depth interviews could allocate sufficient in-depth and first-hand research data; and 2: the data analysis of grounded theoretical research could better construct the corresponding system, analyze the true feelings of marine graduates, and identify the reasons for subjective career unsuccessfulness.

First, participants’ responses were chronologically transcribed and compiled into a coherent document, resulting in a 28,727-word interview transcript for data analysis. Second, the text was read repeatedly to derive categories related to moral knowledge and awareness. Third, according to the steps of open coding, spindle coding, and selective coding of grounded theory, supplemented by the MAXQDA software for coding, the interview records were finally coded and mapped to the corresponding analysis dimensions, and relevant sentences were marked and classified. Analysis continued until no new data or insights emerged.

The coding rules were as follows: the first letter in the code is the gender code (F is for female, M is for male); the second code is for the different participants (represented by numbers 01–12); the third code represents the main category (ZB for adaptation, SY for career adaptability, XD for career action, JG for adaptation result, QJ for situational factor); the fourth code represents the subcategory (indicated by the letter a), and the fifth code is the order of concept expressed (starting from the number 1). Take M4-ZB-a^−1^ as an example; it represents the first concept of the fourth male in the first subcategory of the main category of adaptation preparation.

### Reliability, validity, and research ethics

This study used the triangulation method to ensure the reliability and validity of the analysis of the interview data, including the whole process of audio recording and verbatim translation, interviewing multiple participants in different work times, job positions, and expertise, and recording participants’ key content in the interview process. To enhance the credibility of the study, the initial coding was performed based on the results of the first two respondents. The coding results and all relevant sentences were then extracted to discuss whether the interview analysis and interpretation were reasonable and whether the category was correct or not. When the three experts reached a consensus, the framework and categories of the coding were preliminarily determined, and the coding results of the first two people were revised. After that, the coding of the remaining 10 participants was completed and discussed with the three experts in stages until all three experts reached agreement. The researcher of this study is a teacher of the University of Science and Technology and also a doctoral candidate in education management. Expert 1 is a doctor of vocational education; Expert 2 is a teacher and also a doctoral candidate of the Vocational College of Navigation. These scholars were invited to confirm the credibility of the analytical results and the triangulation of data or methods used correctly to ensure that similar results were obtained (Wang and Wang, 2010).

In terms of validity, participants were allowed to fully express their opinions. The interview outline was provided to the participants 2 h before the interview started, so that they could think about the interview questions in advance. Besides, the proportion of listening, speaking, and questioning was carefully controlled during the interview process to ensure that the participants’ thoughts were fully understood and clarified through timely rhetorical questions to maintain the qualitative research validity (Wolcott, 1994). Finally, in terms of research ethics, the confidentiality of the participants’ data was strictly kept in the process of contacting participants, performing interviews, and analyzing subsequent data analysis.

## Results and discussion

Through repeated reading and comparison of the interview results, the coding was completed according to the steps of open coding, spindle coding, and selective coding as per the grounded theory research paradigm. The coding statistics are shown in Table 3.

**Table 3:** Coding statistics.

Main category	Subcategory	Frequency
Adaptive preparation	Insufficient mental preparation (a)	6
	Lack of determination (b)	7
	Insufficient preparation of certificates (c)	6
	Easy internships (d)	7
Career adaptability	Difficulty of job promotion (e)	6
	Job expectation gap (f)	7
	Boring job (g)	8
	Poor interpersonal relationships (h)	4
	Psychological pressure (i)	5
	Hard work (j)	8
	Higher risk factors (k)	4
	Maladaptation (l)	7
Adaptive action	Job burnout (m)	2
	Lack of setbacks (n)	5
	Lack of competitiveness (o)	9
	Lack of confidence (p)	7
	Lack of short-term plans (q)	8
Situational factors	Bad influence of epidemic (r)	6
	Low social status (s)	2
	Lack of family support (t)	4
	Advantages of work on land (u)	4
Subjective career unsuccessfulness	Hard to make friends (v)	3
	Personality changes (w)	2
	Lack of interest in work (x)	7
	Cannot take care of family (y)	12
	Imbalance between work and family (z)	9
	Salary does not meet expectations (aa)	8
	Bad working environment (ab)	11
	Closed working environment (ac)	8
	Low job achievement (ad)	8

### Adaptive preparation factors

In this study, Adaptive preparation refers to the negative states of weak subjective willingness to adapt or poor readiness to cope with seafarer work. It was concluded from the interviews that the adaptation preparation factors included four parts: lack of psychological preparation, lack of determination and perseverance, lack of certificate preparation, and simple practice work.

According to the coding analysis, six respondents mentioned a lack of mental preparation for a career in seafaring (see below). When they are not mentally prepared, they are anxious, uneasy, have weak subjective psychological willingness to adapt, and have a poor state of preparation because nothing is known while on board. Johnson and Birkeland (2003) also concluded that lack of preparation can lead to career adjustment failure.

I didn't do any preparation; I just got on the boat when they called (M04-ZB-a-1).

Then in fact, I didn't do any preparation for the first boat, but I was still quite nervous. I knew nothing about it … Ah, I was quite anxious (M05-ZB-a-2).

According to the coding analysis, seven participants showed a lack of determination and perseverance (see below). For graduates who converted from school to the new environment of their marine career, their work beliefs were easily disturbed by external factors: the lower the determination and perseverance, the weaker psychological adaptation and psychological preparation, and the more difficult they found it to adapt to the environment. Lamanauskas and Augienė (2014) also concurred that lack of determination and courage interfered with career success.

After playing in Haikou, Hainan for a few days, I started playing wild and didn't want to go back to the ship even though my sailor certificate was renewed (M01-ZB-b-1).

After all, I have stayed at sea for a long time, and if I don't have enough perseverance, I don't think I can stay that long (M07-ZB-b-5).

According to the coding analysis, six participants who did not obtain the certificate of competency for the third mate and the third wheel engineer during their school time faced a problem due to their insufficient preparation for professional competency (as shown below). According to the “Rules of the People’s Republic of China on the Competency Examination and Certification of Sea-Going Seamen” (Order No. 11, 2020 of the Ministry of Transport), “the holder of the certificate of competency shall hold a position or hold a low-level position within the scope of application of the certificate of competency” (The Central People’s Government of the People’s Republic of China, 2020). Insufficient preparation of a professional competency certificate seriously affects job assignments, job promotion, salary raises, and future career development. Furthermore, the tendency of negative professional behavior of seafarers might occur in the long run.

I only have six low-level certificates and a college degree. I do not have the third mate certificate (M01-ZB-c-1).

I failed the test for the third wheel engineer certificate in school, and it is still very difficult to make up the test (M02-ZB-c-2).

According to the coding analysis, seven participants admitted that the internship was easy, as it included only cleaning, knocking off rust, and painting, with no technical content. It represented that the content of the internship only required a little of the knowledge learned in school, giving participants a wrong impression of a marine career; the internship thereby weakened the students’ willingness to continue the internship (Hong et al., 2021).

Monotonous and lacking in technical content. Just repeating those days every day is equivalent to messing around every day (M01-ZB-d-1).

Deckmen, they clean the deck, clean the cabin, do sanitation, rust removal, paint, anchor, stand guard, and watch. Follow the third mate to the bridge on duty. Sailors just do these chores, and there is no technical content (M06-ZB-d-5).

### Career adaptability factors

Career adaptability in this study referred to the psychological ability of graduates to adapt to the marine environment, including recognition of marine work, marine behavior, and emotional regulation. Through the analysis, career adaptability was confirmed to influence the subjective career unsuccessfulness of marine higher vocational graduates including difficulty in career promotion, difference in job expectations, poor interpersonal communication, boring work, hard and busy work, high risk factors, high psychological pressure, and environmental maladaptation.

According to the coding analysis, six participants in this study mentioned difficulties in career advancement (as shown below), so their career development was hindered. They felt worried and disappointed about the future, and even had the idea of giving up their marine career. Career development is the continuous driving force of personal work, focusing on one’s individual future (Savickas and Porfeli, 2012). Paying attention to career development is the ability to judge career adaptation (Wang et al., 2016).

If you haven't taken the third engineer certificate, you won't be in this field, because there is no room for promotion (M04-SY-e-1).

Another is that my promotion is still a long way off (M05-SY-e-2).

According to the coding analysis, seven participants in this study mentioned the problem of the job expectation gap (as shown below). Based on the situational expectation value theory (Eccles and Wigfield, 2020), the expectation before boarding includes high salary, job promotion, and a suitable environment, which are the driving forces for mariner work. However, the actual situation after boarding is totally opposite. If the expectations are not met, the value obviously declines, and then the seafarers perceive a large gap in job expectations that leads to being slack at work, and eventually leaving their jobs.

At that time, I felt that the intensity was very strong, but there was a little drop in expectation (M03-SY-f-2).

The reality is completely different from what was taught in school. It's all gimmicks. It's not as good as I imagined (M08-SY-f-6).

According to the coding analysis, four participants had negative work feelings due to poor interpersonal relationships, due to not being conducive to the normal development of work, and finally leading to career failure (as shown below). Therefore, interpersonal communication skill is an important indicator for evaluating career adaptability (Wang et al., 2016).

Then there are all kinds of gangs, a separation stage, and the things that make me feel very uncomfortable. It is the ability to communicate. I have no way to communicate with these people, so I do it against myself (M05-SY-h-2).

The hierarchy on the ship is still relatively strong. If you can’t stand the ways your superiors talk, even if you get along and communicate with your superiors, you will not be able to do it for long (M07-SY-h-4).

According to the coding analysis, eight participants mentioned being bored on board (see below). A voyage is the maneuvering of a ship by a crew to transport cargo from one place to another. The crews drift on the ocean for a long time in a closed and limited space, doing the same work every day, facing the fixed 20–30 staff on board and the boundless ocean, so they can feel that the work is boring.

But the main point is boredom. It's just a big place, and I just stay there every day, bored and quite idle (M01-SY-g-1).

Too boring, too boring (M03-SY-g-2).

According to the code analysis, eight participants mentioned the problem of hard work and busy work (as shown below). Seafarers are on duty for 6–8 h normally, during which they need to concentrate for a long time, and they often need to increase the frequency of night shifts due to rush loading and unloading of goods. Overtime is required to handle urgent tasks, so rest is seriously affected, and career satisfaction is obviously not high (Carlson et al., 2019).

Because everyone is on duty for eight hours during the voyage; eight hours is quite tiring, because you have to concentrate (M04-SY-j-1).

I was working on a bulk carrier, and I needed to work night shifts when unloading. I was arranged to do a 6-to-6 shift, … 12 hours a day (M12-SY-j-3).

According to the coding analysis, four participants mentioned that there is a high risk factor in the marine profession (as shown below), since spending a long time at sea, encountering emergencies, illnesses, wind and waves, and possibly even pirates all produce uncertainties. It is difficult to obtain timely assistance, and life is often threatened, so it is an occupation with a high risk factor. Moreover, the degree of freedom to control their future career development is not high, and it is largely influenced by the natural environment.

Equipment malfunctions can easily cause chain reactions … I've experienced 1 cabin explosion, 2 engine room fires, the boat was hit, and the boat stalled this situation is dangerous (M06-SY-k-1).

We are definitely a high-risk industry, and it is very inconvenient to get sick on board or encounter some emergencies. So this industry is putting life on the line for a little money (M10-SY-k-3).

And the danger on the boat is particularly high. There were strong winds and waves, and the leader sent me to the deck. The wave hit the back of my butt. It washed things directly off the boat. Another very impressive thing is when crossing the Somali Strait. We were nervous. There should have been pirates, but we didn't encounter them (M12-SY-l-4).

According to the coding analysis, five participants mentioned the problem of psychological stress (see below). Seafarers have been in a limited confined space for a long time, and their freedom is limited; sometimes ships are loaded with dangerous goods, coupled with work fatigue and other reasons. This situation can easily cause great pressure on mariners (An et al., 2020).

There's also a reason that there is high pressure on the boat. I was in a bit of a bad mood at the time (M06-SY-i-2).

Sometimes it is dangerous to pull things on the boat, and there will be psychological pressure (M08-SY-i-3).

Working on a boat is like going to jail; the psychological pressure is too great (M10-SY-i-4).

According to the coding analysis, seven participants mentioned the problem of maladaptation to the environment (as shown below), and the unsuitable diet seriously worsened the crew. Some participants also expressed that they were troubled by seasickness, which had a very severe impact on their career experience.

When the chef's cooking is not to my taste, it is actually a negative situation (M05-SY-l-1).

I feel that when the typhoon is very serious, and I get seasick but I have to continue to work, no one can help me or replace me. I feel very helpless, and I really vividly remember it (M06-SY -l-2).

### Adaptive action factors

The study results showed that adaptive actions referred to the negative reactions, choices, and behaviors of graduates after completing self-adjustment in adapting to marine work. The adaptive action factors affecting the subjective career unsuccessfulness of marine vocational graduates were: job negativity and burnout, lack of frustration and training, lack of strong competition, lack of confidence in development, and lack of recent planning.

According to the coding analysis and the situational expectation value theory (Eccles and Wigfield, 2020), two participants showed negative job burnout (as shown below), were unwilling to continue working, and felt bored. The expectations of marine graduates for work on board are mainly high salary, which is the only motivation for many participants to work hard. However, the actual income may be lower than expected, or they may not be promoted for a long time, so they will feel that the value is reduced or diminished. Working at sea also requires separation from family members, disconnection from society, and being unable to enjoy life and take care of their family. As a result, a lack of motivation leads to negative actions, such as job burnout, lack of planning in recent years, or lack of confidence in development. As Hassan and Ibourk (2021) mentioned, the higher the level of job burnout, the lower the job satisfaction and the more likely they are to have negative feelings.

Then I learned to be lazy in the middle, and then I figured out how to quit my job later. I lost the energy when I was doing it, and I felt tired, so I didn’t want to work anymore (M08-XD-m-1).

I don't like the job or the crew so much. I have lived on the ship for a year. I am tired of that life and don't want to go on board again (M12-XD-m-2).

According to the coding analysis, five participants mentioned that they did not encounter many setbacks or difficulties in their careers (as shown below). Even though the voyage was hard, the seafarers faced isolation from society and outsiders, life became relatively simple, and the problems and difficulties encountered were relatively minor to many participants.

I have not encountered any setbacks or difficulties in my work (M02-XD-n-1).

It seems that I have never encountered any setbacks or difficulties at work. If you don't understand something, just ask your leader; as long as you don't do anything wrong (M07-XD-n-3).

According to the coding analysis, nine participants pointed out a lack of strong competition on the ship (as shown below), because most of the ships have one person in each position with no competition. As mentioned by Spurk et al. (2021), competitive psychology is negatively related to job satisfaction through burnout. In other words, job burnout and lack of competition lead to a decrease in job satisfaction.

Not competitive. No, I didn't experience competitiveness. (M04-XD-o-2).

Not competitive (M12-XD-o-9).

According to the coding analysis, seven participants said they did not have enough confidence in their career development, showing confusion and frustration.

I don't know about my career development and feel lost. Taking the college certificate as an example, I need to make up the exam, and I haven't studied enough, so I really lack confidence in taking the college certificate (M02-XD-p-1).

Hey, what can I say about my confidence in my own career development, I don't have any hope, and I am disheartened (M06-XD-p-3).

According to the coding analysis, eight participants said that they had not made long-term plans for their marine career in recent years (as shown below), or had only made short-term plans for 1–2 years, or even within a few months, because they all thought that they did not consider working onboard as their life-long career. Proper planning is the key to success (Zwikael et al., 2014); on the contrary, lack of planning can easily lead to failure.

I was thinking about doing it for 6 months after the internship, and then quitting after 6 months (M04-XD-q-3).

I didn't think that far, and was disheartened. I just want to work for 2 months and go home to repay the debt, and then find something to do at home (M10-XD-q-6).

### Situational factors

The study results showed that the situational factors that influenced the subjective career unsuccessfulness of marine vocational graduates include: adverse effects of the epidemic, lack of attention from society, lack of support from family members, and the advantages of working on land.

According to the coding analysis, six participants mentioned that the maritime industry was deeply affected by the COVID-19 epidemic. The seafarers were trapped on the ship for a long time because of the epidemic. Some participants said that it was like living in prison and they were afraid of not getting off the ship. Based on the situational expected value theory (Eccles and Wigfield, 2020), the crew re-evaluated the expected value of the situation due to the impact of the epidemic. Although they receive high pay as remuneration for their work onboard, they are at risk of contracting new contagious diseases. The cost of working onboard is too high compared with land jobs; on land, even though the pay is less, they can stay safe.

Because of the impact of the epidemic, I disembarked in 2020, and I didn't go back since then (M09-QJ-r-3).

The ship is like a moving cage. Now, due to the impact of the epidemic, the crew is not allowed to disembark. The ship is more like a moving water prison (M11-QJ-r-5).

According to the coding analysis, two participants talked about the low social status of seafarers (as shown below). People tend to think that seafarers work in a high-risk industry in exchange for high wages. As Lee et al. (2021) said, low social status indicates that the society has less emotional care for seafarers. Due to this phenomenon, seafarers with stronger career adaptability are more likely to leave. For companies, giving more emotional care to the seafarers, improving their social status, paying more attention to their health, ensuring considerable salary compensation, ensuring normal working hours, advocating a positive organizational culture and values, and so on, can effectively improve the working conditions of seafarers and enhance their life satisfaction (Chen et al., 2020).

Neither society nor the country has great concern for the seafarers. Former United Secretary-General Ban Ki-moon said that if there were no crew seafarers in the world, half of the people would be comfortable but half would go hungry. But few really pay attention to seafarer development (M06-QJ-s-1).

The social status of seafarers is particularly low, and it is a very unseemly job. … Everyone looked at me strangely … people look down on seafarers, … they think that you are an industry with no technical content at all, and it is nothing more than a high-wage industry in exchange for high risks (M10-QJ-s-2).

According to the coding analysis, four participants agreed that the lack of family support also seriously affected the career development of marine graduates (see below). Guan et al. (2016) pointed out that parental support had a significant positive impact on college students’ career adaptability, whereas lack of parental support can lead to poor career adaptability of students.

I worked hard to earn money on the boat. In addition to being scolded and complained to by my girlfriend, I was not understood by my girlfriend. I was also very angry, and then we broke up (M06-QJ-t-2).

The ideological work of the family and the ideological work of the girlfriend have not been done well. I didn't introduce my job in detail to my parents, which caused my family members to worry and they wanted me to come back and have a stable job on land (M07-QJ-t-3).

According to the coding analysis, four participants said that with the development of China’s economy in recent years, the country has become richer and stronger, and the advantages of free work on land, taking into account the family, good salary, and more development opportunities have become increasingly prominent. Due to the disadvantages of being closed off and separated from society, more marine graduates give up their jobs on ships and choose jobs on land instead.

I feel that the money earned on shore and on the boat is basically the same, but it is better on land. Although the consumption is relatively high, it is freer, and you can go wherever you want (M01-QJ-u-1).

Therefore, if you open your own store on land, the income will be higher. Also, I feel freer on land (M08-QJ-u-3).

It was not as good as my current job on land. I earn seven or eight thousand yuan a month and I don't have to stay in one place all the time, and I can have fun (M12-QJ-u-4).

### The connotation of subjective career unsuccessfulness

According to the coding analysis, the adaptation result of this study was that the graduates have poor interaction and integration with the marine work environment, resulting in negative personal career feelings and subjective career unsuccessfulness. Specifically, it referred to work-life imbalance, a low sense of work achievement, poor work environment, closed work environment, unsatisfactory salary, lack of interest in work, personality changes, and difficulties in their social life.

## Conclusion and recommendations

### Conclusion

To effectively promote the career management and counseling of marine students, the enhancement of job adaptability and career satisfaction after graduation can avoid the situation of subjective career unsuccessfulness. Therefore, qualitative research was adopted, through in-depth interviews with marine graduates to understand their personal feelings about engaging in marine-related careers. Adopting the career construction theory and situational expectation value theory helps to attribute the factors affecting graduates’ subjective career unsuccessfulness. In general, the 12 participants held a negative attitude towards their personal marine career and they subjectively believed that their careers were unsuccessful.

The factors of adaptability and preparation affecting the career unsuccessfulness of marine graduates in higher vocational colleges are psychological preparation, determination and perseverance, lack of certificate preparation, and the simplicity of the internship work. Influencing factors for career adaptability are boring jobs, busy work, an expectation gap, large coefficient of risks at work, difficulty of career promotion, poor interpersonal relationships, high psychological pressure, and poor adaptability to the environment. The adaptive action factors are negative work burnout, lack of coping with frustration and lack of strong competition, lack of confidence in development, and lack of planning in recent years. The situational factors are the adverse effects of the epidemic, low social status, insufficient family support, and the advantages of land work.

This study indicated that mental preparation, determination perseverance, insufficient preparation for certification, and the simplicity of internship work were the four influencing factors. This concurred with the statements of Johnson and Birkeland (2003) who pointed out the lack of preparation, and Lamanauskas and Augienė (2014) who mentioned the lack of determination and courage that would interfere with the achievement of career success. Hong et al. (2021) also mentioned that the content of the internship had little to do with the knowledge learned in school, which gave the interns the feeling that the internship was useless and thus weakened their willingness to continue with it. All of these research results were similar to the findings of the current study. The other two influencing factors of insufficient family support and greater psychological pressure were similar to Bao et al. (2021) finding that family responsibility and health status was the main factor leading to the exodus of young seafarers. A clear career plan to become senior officials was not stable due to the lack of planning in recent years, leading to subjective career unsuccessfulness. An et al. (2022) and Tetemadze et al. (2021) argued that excessive workload, insufficient sleep, and fatigue affected ship safety. In addition, Tetemadze et al. (2021) stated that lack of on-board socialization and poor interpersonal relationships caused crew fatigue and worsened seafarer health.

Besides, the adverse effects of the epidemic, poor interpersonal relationships, and hard work mentioned in this study were similar to those mentioned by Carrera-Arce et al. (2022). COVID-19, factors related to rest and leisure time, communication/relationships with the outside world, and onboard interactions were the factors in seafarers’ social life which affected their mental health and well-being. Brooks and Greenberg (2022) proposed that sailing was often acknowledged as a dangerous occupation, so crew members felt unsafe, and there was poor social support, which supported the results of this study indicating a high risk factor and poor interpersonal relationships. Beukelaer (2021) and Okeleke and Aponjolosun (2020) confirmed that COVID-19 made crew shift change difficult and had a severe impact on the shipping industry, causing seafarers to be mentally and psychologically distressed and to have low job satisfaction. McVeigh et al. (2019) pointed out that a supportive work environment affects seafarers’ work experience. This study found that environmental maladaptation led to subjective career unsuccessfulness.

Due to repetitive work in a confined environment, crew members felt bored, lacked strong competition, and manifested work passivity and burnout. Some participants in this study felt that career advancement was difficult and they lacked confidence in their career development because they had not yet obtained qualification certificates such as third mate and third officer. Moreover, when the students were aware of the gap between salaries actually paid and promised before entering the industry, and the working environment and salary treatment were not as good as they had been led to believe, an expectation gap occurred. In addition, the advantages of working on land were prominent, and the salary gap between the on-land crew and the seafarers was narrowed. Therefore, more nautical graduates were more inclined to choose to work on land.

### Recommendations

For students majoring in navigation, considering the difficulties they face in their career development, the school needs to strengthen the work in the following aspects.

Strengthen students’ mental health education as followings: 1. guide students to correctly understand the development and employment of the maritime profession; 2. analyze the pros and cons of different employment directions; 3. prepare students psychologically in advance; 4. cultivate students’ determination to overcome difficulties and meet challenges; 5. exercise their perseverance and tenacity; 6. teach students to relieve their psychological pressure; 7. build confidence in their career development. Then, guide students to find out the meaning of simple work and enhance the experience of work achievement; teach students to accept the dullness of work with a peaceful mind, and establish a way to adjust to the boring life; broaden students’ interests and hobbies; delve into, share, and pass on interesting things in life. Appropriate fun activities on board can also help seafarers reduce their fatigue and stress (Gu et al., 2020).

Guide students to make long-term career planning. Students are constantly urged and encouraged to obtain more vocational competency certificates, consolidate their professional knowledge and skills, and enhance their competitiveness.

Strengthen students’ language communication and interpersonal skills. Effective communication can facilitate the smooth development of work. Schools can offer courses related to interpersonal communication, or promote students’ language communication and interpersonal skills by establishing clubs and organizing activities.

Strengthen the physical exercise of students and enhance their safety education. Almost all seafarers experience seasickness, and often encounter wind and waves, and even pirates. As a result, the safety of seafarers is often threatened, which makes working onboard a high-risk occupation. To fulfill the occupational requirements more quickly and effectively, students must have a strong physique and a high level of safety awareness.

Strengthen school-industry cooperation and the supervision of company recruitment. This study showed that the chaos caused by bad management of ships also has a negative effect on the seafarers’ work experience. Schools need strong supervision of the companies and should ensure that the recruiting companies are of high quality so that the students’ basic interests are not infringed.

### Research significance

This study has theoretical significance as follows: first, this study firstly proposed subjective career unsuccessfulness and determined its connotation, which enriched the research on career development through the method of qualitative research. Second, by supplementing the theoretical framework of career construction, the analysis explained the factors of subjective career unsuccessfulness of nautical graduate students and stated various factors affecting career unsuccessfulness in each link of career construction, which enriched the career construction theory. Third, this study used the contextual expectation value theory to assist in explaining career unsuccessfulness, which effectively promoted the application of the theory.

### Research limitations and suggestions for future studies

This study systematically analyzed the influencing factors of subjective career unsuccessfulness of marine navigation graduates in higher vocational colleges through grounded theory, which has a certain reference value for promoting the career development and educational practice of marine navigation students in the future. The chosen school had nine graduates of marine navigation, which was a typical representation in China. However, there are still some limitations in this study: 1. although the participants in this study are representative of nautical students, they were all from the same vocational undergraduate college. Therefore, the scope of the study can be expanded in the future to interview graduates from different colleges. 2. The concept of subjective career unsuccessfulness was first proposed by the essential studies, which is a relatively new concept. In the future, quantitative research methods can also be used to evaluate the perceptions of participants. 3. The participants in this study were only graduates of the marine category. In the future, research on students of different majors can be carried out to expand the similarities and differences of subjective career unsuccessfulness in different industries and discuss the influencing factors of different industries.

## Data availability statement

The raw data supporting the conclusions of this article will be made available by the authors, without undue reservation.

## Ethics statement

Ethical review and approval were not required for the study on human participants in accordance with the local legislation and institutional requirements. The patients/participants provided their written informed consent to participate in this study.

## Author contributions

LW, J-HY and Y-SL: concept and design and drafting of the manuscript. LW, J-HY and C-JM: acquisition of data, Grounded theory coding and analysis. LW, J-HY and Y-SL: critical revision of the manuscript. All authors contributed to the article and approved the submitted version.

## Funding

This work was financially supported by the Key Projects of Teaching Reform of Education Department of Hainan Province, China (Exploration and Research on the Essential Connotation and Characteristic Development of Vocational Undergraduate, No. Hnjg2021ZD-51).

## Conflict of interest

The authors declare that the research was conducted in the absence of any commercial or financial relationships that could be construed as a potential conflict of interest.

## Publisher’s note

All claims expressed in this article are solely those of the authors and do not necessarily represent those of their affiliated organizations, or those of the publisher, the editors and the reviewers. Any product that may be evaluated in this article, or claim that may be made by its manufacturer, is not guaranteed or endorsed by the publisher.
